# Interaction of HTLV-1 Tax with minichromosome maintenance proteins accelerates the replication timing program

**DOI:** 10.1186/1742-4690-8-S1-A140

**Published:** 2011-06-06

**Authors:** Mathieu Boxus, Jean-Claude Twizere, Sébastien Legros, Richard Kettmann, Luc Willems

**Affiliations:** 1 National Fund for Scientific Research, Gembloux Agro-Bio Tech, Cellular and Molecular Biology, University of Liège, Gembloux, 5030, Belgium; 2 National Fund for Scientific Research, Interdisciplinary Cluster for Applied Genoproteomics (GIGA), University of Liège, Liège, 4000, Belgium

## 

The Tax oncoprotein encoded by the Human T-cell leukemia virus type 1 (HTLV-1) plays a pivotal role in viral persistence and pathogenesis. HTLV-1 infected cells proliferate faster than normal lymphocytes, expand through mitotic division and accumulate genomic lesions. Here, we show that Tax associates with the minichromosome maintenance MCM2-7 helicase complex and localizes to origins of replication. Tax modulates the spatiotemporal program of origin activation and fires supplementary origins at the onset of S phase. Thereby, Tax increases the DNA replication rate, accelerates S phase progression but also generates a replicative stress characterized by the presence of genomic lesions. Mechanistically, Tax favors p300 recruitment and histone hyperacetylation at late replication domains advancing their replication timing in early S phase.

